# Post-HSCT graft failure due to refractory human cytomegalovirus successfully treated with haploidentical donor-derived immunoglobulins and stem cell graft infusion: A case report

**DOI:** 10.1016/j.antiviral.2021.105024

**Published:** 2021-04

**Authors:** Francesco Baldo, Nicolás M. Suárez, Andrew J. Davison, Davide Zanon, Egidio Barbi, Natalia Maximova

**Affiliations:** a Department of Medicine, Surgery and Health Sciences, University of Trieste, Trieste, Italy; b MRC-University of Glasgow Centre for Virus Research, Glasgow, United Kingdom; c Institute for Maternal and Child Health – IRCCS Burlo Garofolo, Trieste, Italy

**Keywords:** Human cytomegalovirus, Post-transplant graft failure, Targeted immunotherapy, Hyperimmune plasma infusion

## Abstract

**Background:**

Human cytomegalovirus (HCMV) remains an important cause of transplant-related morbidity and mortality. The incidence of HCMV recurrence in the donor seronegative (D-)/recipient seropositive (R+) group is significantly higher than in other serostatus combinations as a result of a lack of pre-existing HCMV-specific memory T-lymphocytes in the donor, coupled with the eradication of the recipient's cellular immunity due to the conditioning regimen.

**Case presentation:**

We describe the case of an 8-year-old βE-thalassemic girl from Bangladesh who was seropositive for human cytomegalovirus (HCMV) and underwent hematopoietic stem cell transplantation from a HLA-matched, unrelated, HCMV-seronegative donor. Despite administering antiviral prophylaxis with commercial pooled anti-HCMV immunoglobulin (Ig) from day +1, the post-transplant course was complicated by prompt viral reactivation, and foscarnet therapy was initiated. The virus was refractory to treatment, leading rapidly to complete bone marrow failure, and targeted immunotherapy was proposed as a second-line therapy. Hypothesizing that the patient and her relatives may have been exposed to similar HCMV strains, we selected the patient's mother, who presented a high HCMV antibody titer, as the donor of virus strain-specific anti-HCMV Ig and T-lymphocytes. Complete viral clearance was achieved after two transfusions of the mother's plasma. Subsequently, the patient underwent a haploidentical rescue transplant, promptly reaching full hematological recovery.

**Conclusion:**

These findings suggest that treatment with virus strain-specific Ig may offer a new therapeutic option for critically ill patients.

## Highlights

• Human cytomegalovirus (HCMV) remains an important cause of transplant-related morbidity and mortality.
• Reactivation of multiple viral strains is particularly challenging to eradicate. .
• Virus strain-specific Ig is an inexpensive, non-toxic therapy to treat HCMV reactivation and improve transplant outcomes. .
• The efficacy of this approach as a means of prevention has only been demonstrated previously in mouse models.

## 1 Introduction

Human cytomegalovirus (HCMV) is the most significant cause of opportunistic viral infection in allogeneic hematopoietic stem cell transplantation (HSCT) and remains an important cause of transplant-related morbidity and mortality (Wagner-Drouet et al., 2019). Several studies have shown that the incidence of HCMV recurrence in the donor seronegative (D-)/recipient seropositive (R+) group is significantly higher than in other serostatus combinations (Ganepola et al., 2007; Zhou et al., 2009; Ozdemir et al., 2007). This is likely a consequence of delayed HCMV-specific immune reconstitution due to the lack of pre-existing HCMV-specific memory T-lymphocytes in the donor, coupled with the eradication of the recipient's cellular immunity due to the conditioning regimen (Styczynski, 2018; Ljungman et al., 2014). HCMV infection can lead to pneumonia, gastroenteritis, retinitis, hepatitis, and encephalitis among HSCT recipients (Griffiths, 2012). In addition, HCMV infection of the bone marrow can result in impaired graft acceptance and, in the most extreme cases, complete graft failure (Steffens et al., 1998). Treatment of HCMV disease essentially consists of administering antiviral drugs, namely ganciclovir and foscarnet, and intravenous immunoglobulin. Although chemoprophylaxis is not generally recommended in HSCT, immunoglobulin, whether pooled or HCMV-specific, have shown some efficacy in preventing recurrent HCMV infections (Boeckh and Ljungman, 2009).

We describe a case of a pediatric HSCT recipient who had complications of HCMV recurrence and was treated successfully with targeted immunological therapy, using haploidentical donor-derived immunoglobulin (Ig).

## 2 Case presentation

An 8-year-old girl was diagnosed at the age of two in her country of origin (Bangladesh) as βE-thalassemic. She was treated with monthly blood transfusions and weekly iron chelation with deferoxamine, plus supplementation with folic acid. A suboptimal regimen of chelation therapy led to severe systemic siderosis, and her family travelled to Italy to undergo HSCT. Since a human leukocyte antigen (HLA)-matched related donor was unavailable at that time, an HLA-matched (10/10) unrelated donor was identified. The patient was HCMV-seropositive, whereas the donor was HCMV-seronegative, thus posing a high-risk serological combination (D-/R+) for post-transplant HCMV infection and associated complications.

The conditioning regimen consisted of fludarabine, busulfan, thiotepa, and anti-thymocyte globulin (ATG). The infused donor's bone marrow contained 3.79 × 10^8^ total nuclear cells/kg recipient weight, and the vitality of the cells contained in the graft was 96.9%. Tacrolimus and mycophenolate mofetil were used for graft-versus-host disease (GVHD) prophylaxis. Anti-HCMV prophylaxis commenced at day +1 with commercial pooled anti-HCMV Ig (Megalotect, Biotest Pharma GmbH, Germany) at a dose of 100 U/kg twice a week.

The initial post-transplant period was uneventful. The first occurrence of HCMV viremia (600 viral genome copies/mL) was detected at day +14, and antiviral therapy with foscarnet was started immediately. At day +17, neutrophil engraftment was achieved, but not the platelet and the erythrocyte counterparts. At day +20, the patient presented fever without clinical symptoms and without an increase of C-reactive protein. Afterwards, a full blood count revealed a progressive drop in leukocytes and platelets, whereas HCMV viremia continued to increase despite antiviral therapy. The patient also developed a severe, HCMV-related hemorrhagic cystitis, which required frequent platelet transfusions, initially once daily for 5 days, and thereafter twice daily for 7 days.

As the infection appeared unresponsive to foscarnet, ganciclovir treatment was started at day +25. This switch in antiviral therapy followed an increase in viral load, reaching a maximum titer of 5100 viral genome copies/mL at day +30. Simultaneously, the clinical condition of the patient continued to deteriorate, and the leukocyte count decreased drastically. Therefore, the diagnosis of secondary graft failure due to HCMV infection was established. Because of the failure of anti-HCMV treatment, ganciclovir was withdrawn, and foscarnet therapy was resumed. The possible explanations for treatment failure include the presence of antiviral resistance mutations or an ineffective activity of the Ig administered. As the first option seemed improbable because resistance mutations usually arise later than 6 weeks after HSCT, we opted to address the latter option. Therefore, we considered using antibodies (Abs) that were more likely to inhibit the HCMV strains causing the infection in the patient. Although the diversity of circulating HCMV strains is known to be extensive (Puchhammer-Stöckl and Görzer, 2011), we hypothesized that the patient and her relatives may have been exposed to similar strains on a familial basis. As a result, we performed serological tests on the patient's mother, father and brother. We selected the patient's mother, who presented the highest anti-HCMV IgG titer, as the best donor of strain-specific HCMV Ab-rich plasma and virus-specific T-lymphocytes in this situation. At day +32, the patient's mother underwent leukapheresis. The lymphocytes were sent immediately to a cell factory to produce specific anti-HCMV T-lymphocytes. On the same day, the plasma enriched in HCMV strain-specific Abs was infused into the patient. At day +34, the viral load decreased for the first time, to 1100 viral genome copies/mL, and the patient began new conditioning for a haploidentical rescue transplant from her mother. In light of the concurrent bone marrow aplasia, the conditioning regimen was conducted solely with ATG. At day +38, the girl received a second plasma transfusion from her mother. On the day of the second transplant (day +40), complete viral clearance was documented, and the girl received 11.3 × 10^6^ CD34 cells/kg of mother's graft. GVHD prophylaxis was performed with post-transplant cyclophosphamide, tacrolimus, and mycophenolate mofetil. The engraftment was rapid, with a neutrophil count of >500/μL achieved at day +15, and the final platelets and packed red blood cells were transfused at day +20. Since HCMV viremia remained negative during the entire post-transplant period, we discarded the use of the mother's virus-specific T-lymphocytes. The patient was discharged at day +27 in perfect clinical condition, with continuation of antiviral valganciclovir prophylaxis. Immunosuppressive treatment and antiviral prophylaxis were discontinued six months later, and the patient remains in perfect health 2 years after the second transplant.

**Fig. 1:**
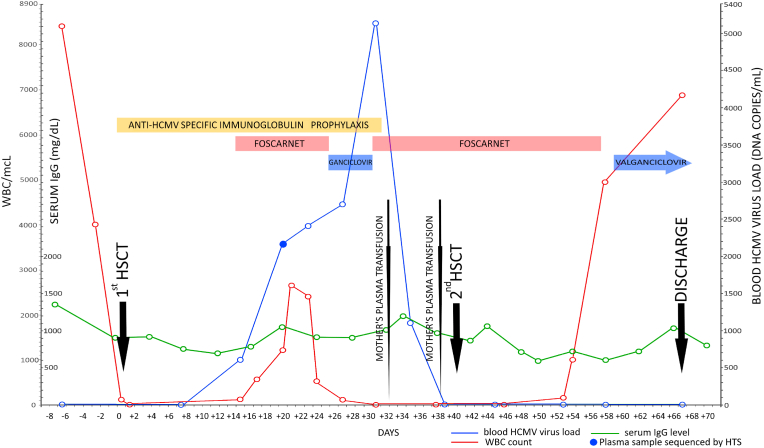
Information on viremia, white blood cell (WBC) count, total serum IgG and antiviral therapy. Data on HCMV viremia is expressed in viral genome copies/mL (right Y-axis). Application and duration of antiviral treatments are represented by colored bars (yellow, anti-HCMV-specific Ig; red, foscarnet; blue, ganciclovir/valganciclovir). Chronological data (X-axis) are represented in days after the first transplant. The plasma sample analysed by high-throughput sequencing (HTS) is represented by a filled blue circle.

Therapeutic interventions, HCMV load, and white blood cell count during the entire episode are summarized in Fig. 1. In addition, the HCMV strains present in the patient were characterized by high-throughput sequencing (HTS) of an archived (−80 °C) plasma sample from day +19. DNA was extracted from 800 μl of plasma using a QIAamp MinElute virus spin kit (QIAGEN, Crawley, UK). An aliquot of 50 μl of DNA was used to generate an HCMV-enriched sequencing library as described previously (Suárez et al., 2019). The library was loaded onto a NextSeq DNA sequencer (Illumina, San Diego, CA, USA), generating 150 bp paired-ended reads (Table 1). HCMV strain enumeration was performed by genotyping 12 hypervariable HCMV genes from the reads as described previously (Suárez et al., 2019). The detection of four genotypes of the RL13 gene indicated the presence of at least four HCMV strains (a strain being defined as a particular constellation of genotypes of the hypervariable genes analysed), each including one of these RL13 genotypes (Table 2). The data was also inspected for the presence of resistance mutations in the antiviral target genes (UL54 and UL97) using LoFreq (Wilm et al., 2012). Briefly, the reads were mapped to the genome of HCMV reference strain Merlin (GenBank accession no. AY446894.2), and non-synonymous variants known to confer resistance were identified. No resistance mutations were detected.

**Table 1:** Overview of HTS data.

Sample ID	TRI-SCT1
Sample type	Plasma
Days after transplant	+19
HCMV load (genome copies/mL)	2100
Data deposition (ENA project no.)^a^	PRJEB38333
Sequencing library (genome copies)	1440
Trimmed reads (no.)	5,033,480
HCMV reads (no.)^b^	936,773
HCMV reads; %^b^	19
Coverage depth (reads/nt)^b^	885

a The sequence dataset was purged of human reads and deposited in the European Nucleotide Archive (ENA).

b Reads mapped to the HCMV reference strain Merlin genome; HTS, high-throughput sequencing; nt, nucleotide.

**Table 2:**
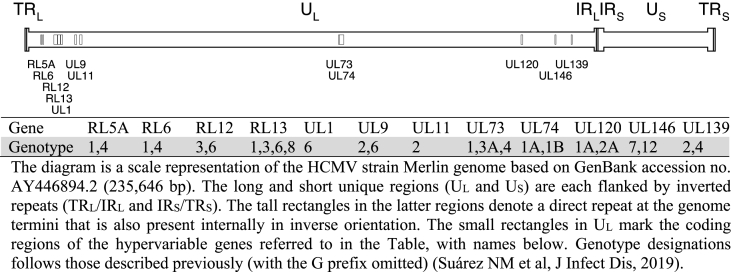
Genotyping of hypervariable HCMV genes.

## 3 Discussion

Various factors are implicated in the outcome of HCMV infection in transplant patients, the first of which relates to the HCMV serological status of donor and recipient. The serological status of the first donor may have played an important role in this case, as it has been reported that using an HCMV-seronegative unrelated HSCT donor (D-) for a seropositive recipient (R+) results in reduced overall survival after myeloablative conditioning. In contrast, an HCMV seropositive donor is associated with improved event-free survival and reduced non-relapse mortality (Styczynski, 2018). Thus, the absence of an HCMV-specific immunological memory in the donor's cells may have placed the HCMV-positive recipient at a higher risk of HCMV reactivation and associated complications.

In relation to viral factors, the clinical course of the patient may have been influenced by two main factors: the presence of multiple HCMV strains or the presence of mutations conferring antiviral resistance. Infection with multiple HCMV strains is common after transplantation (Görzer et al., 2010), and is associated with delayed HCMV clearance during antiviral therapy, even in the absence of antiviral resistance mutations. It is also associated with worse clinical outcomes, including an increase in graft rejection and faster progression of disease. In principle, the mechanisms behind this increased pathogenicity can be viral, for example involving recombination and complementation of different strains within the host, or immunological, since control of multiple strains may be more challenging for the immune system. Overall, in clinically symptomatic immunocompromised hosts, the presence of multiple HCMV strains is more threatening than the presence of a single strain (Manuel et al., 2009).

In the present study, the initial failure of antiviral therapy could not be explained by the development of resistance mutations in the HCMV strains present in the patient. This finding is not surprising, as detection of resistance mutations is unusual during the first 6 weeks of antiviral therapy, whereas the sample analysed in this case was collected only 13 days after initiation of foscarnet treatment (Lurain and Chou, 2010; Springer et al., 2005).

In regard to the initial failure of the immunotherapy with commercial pooled Ig, this may have been due to the lack of particular Abs with neutralizing capacity against the HCMV strains present in the patient. Assuming that the donors providing the Ig had been exposed to strains circulating in Europe, the Abs may have been more suited for neutralizing HCMV strains commonly circulating in that region. However, it is possible that the strains present in the patient, who originated from Bangladesh, may have differed immunologically from strains in other regions of the world. The extent to which this is true is unknown, as current knowledge of HCMV genome variability is derived almost entirely from strains circulating in a few European countries. Therefore, it would be useful to assess the neutralizing capacity of commercial pooled Ig in order to avoid potential therapeutic failure, especially in patients from non-European countries. The clinical problem in this case was addressed successfully by using the Ab-rich plasma of the patient's mother in a kind of personalized immunotherapy, on the premise that both mother and daughter may have been exposed to the same pool of viral strains. In this HCMV-positive recipient with a severe viral reactivation, Ig obtained from a household donor afforded an efficacious treatment that led to complete viral clearance.

To the best of our knowledge, this is the first report of the successful use of HCMV strain-specific Ig therapy in an HSCT recipient. The efficacy of this approach as a means of prevention has been demonstrated previously only in mouse models involving murine cytomegalovirus (MCMV) reactivation after bone marrow transplantation (Martins et al., 2019). In this setting, mice received serum obtained either from latently infected (seropositive) donors or from seronegative donors. The transfer of immune serum protected mice from viral reactivation without affecting the development of GVHD. Remarkably, complete viral protection was obtained with a small volume (5 μL) of strain-specific immune serum. On the other hand, when specific donor antibodies were administered to mice infected with antigenically mismatched MCMV strains, they were not efficient in preventing viral reactivation.

Our case highlights the importance of monitoring HSCT recipients comprehensively, longitudinally and with high sensitivity for viral factors, including the presence of multiple strains and resistance mutations. Importantly, it also provides an affordable targeted immunological therapy in patients from countries with constrained access to alternative therapies.

## 4 Conclusion

HSCT is a complex and specific setting in which a recipient may rapidly develop viral reactivation and consequent life-threatening complications. Ab-rich plasma may represent a fast and feasible therapeutic option to overcome viral activity and allow successful engraftment of the donor's bone marrow.

## Funding

No specific funding was received.

## Consent for publication

The authors have obtained consent from the parents of the patient to publish individual patient data.

## Declaration of competing interest

The authors declare that they have no known competing financial interests or personal relationships that could have appeared to influence the work reported in this paper.
